# *Drechslerosporium cornellii* gen. et sp. nov. within the Basidiobolaceae exhibiting unique conidial discharge and digitate chlamydospores

**DOI:** 10.3897/mycokeys.136.200461

**Published:** 2026-07-07

**Authors:** Yong Nie, Bo Huang

**Affiliations:** 1 Anhui Provincial Key Laboratory for Microbial Pest Control, Anhui Agricultural University, Hefei 230036, China School of Civil Engineering and Architecture, Anhui University of Technology Ma’anshan China; 2 School of Civil Engineering and Architecture, Anhui University of Technology, Ma’anshan 243002, China Anhui Provincial Key Laboratory for Microbial Pest Control, Anhui Agricultural University Hefei China

**Keywords:** Morphology, new taxa, phylogeny, systematics, zygomycetes

## Abstract

We describe *Drechslerosporium cornellii***gen. et sp. nov**., a phylogenetically distinct fungal genus isolated from decaying plant litter at Cornell Plantations, Ithaca, New York, USA. This novel monotypic genus is assigned to the family Basidiobolaceae and is characterized by two unique morpho-developmental traits: (1) retention of prominent conidiophore appendages on discharged conidia following circumscissile rupture of the apical swelling and (2) direct ontogeny of digitate chlamydospores from primary conidia, a trait unrecorded in related genera. Multilocus phylogenetic analysis of nuclear ribosomal 28S, ITS, and *RPB2* sequences robustly resolves Drechslerosporium as a sister lineage to *Basidiobolus* within Basidiobolaceae, yet forming a distinct evolutionary clade. This phylogenetic placement, coupled with its unique morphological characteristics, necessitates formal recognition of *Drechslerosporium* as an independent genus. Accordingly, a revised taxonomic circumscription of the Basidiobolaceae is provided to accommodate the newly discovered phenotypic and phylogenetic diversity. These findings clarify specialized discharge mechanisms driving spore dispersal evolution and expand our understanding of structural diversification within early-diverging lineages of fungi.

## Introduction

The genus *Basidiobolus* Eidam, established in 1886 with *B. ranarum* as the type species, comprises ecologically versatile fungi with a cosmopolitan distribution in soil, decaying organic matter, and amphibian feces, typically disseminated through arthropod vectors (Drechsler 1964; Gugnani and Okafor 1980; Sitterlé et al. 2017). Primarily recognized as saprotrophs in natural ecosystems and historically as animal (herptile) symbionts, several *Basidiobolus* species have also caused subcutaneous and systemic infections in humans, raising clinical and epidemiological concerns (Al-Hatmi et al. 2021).

*Basidiobolus* is morphologically defined by two distinctive characteristics: (1) uninucleate conidia forcibly discharged through a “conidial rocket” mechanism—a process wherein pressurized expulsion of conidiophore cytoplasm drives conidial propulsion following circumscissile rupture of the apical distention—and (2) the production of thick-walled zygospores serving as resting structures (Humber 2012).

Traditionally, *Basidiobolus* has been classified as the sole genus of the monogeneric family Basidiobolaceae within the order Entomophthorales (Ben-Ze’ev and Kenneth 1982; Humber 1989). However, molecular phylogenetic investigations have fundamentally challenged this taxonomic framework. Early rDNA-based studies demonstrated that *Basidiobolus* exhibits significant phylogenetic divergence from other Entomophthorales, instead grouping with Chytridiomycota representatives (Nagahama et al. 1995; Jensen et al. 1998; Cavalier-Smith 1987, 1998; Tanabe et al. 2000, 2003; Keeling 2003; Lutzoni et al. 2004; James et al. 2006). Subsequent multilocus phylogenomic analyses, incorporating data from diverse genomic regions and large-scale proteomic datasets, have yielded inconsistent topologies regarding *Basidiobolus*’ phylogenetic position and higher-order classification, resulting in unresolved placement across multiple taxonomic hierarchies (Spatafora et al. 2016, 2017; Davis et al. 2019; Wang et al. 2020; Galindo et al. 2021; Strassert and Monaghan 2022; Wang et al. 2023; Carleton et al. 2026). Despite these persistent molecular ambiguities, the monogeneric status of Basidiobolaceae has remained entrenched in mycological taxonomy. For the purposes of this study, we adopt Humber’s systematic classification framework, positioning *Basidiobolus* within the family Basidiobolaceae, order Basidiobolales, class Basidiobolomycetes, and phylum Entomophthoromycota (Humber 2012).

During a targeted mycological survey of *Conidiobolus* and *Basidiobolus* diversity in Ithaca, New York, we isolated an undescribed fungal strain from plant detritus. This isolate shares key morphological synapomorphies with *Basidiobolus*, including conidiophores with apical swellings and forcibly discharged uninucleate conidia via conidiophore cytoplasmic expulsion. However, it exhibits several critical morphological divergences: (1) retention of a basal membranous appendage derived from the conidiophore apex post-discharge, never separating from the conidium, (2) a distinct positioning of the circumscissile rupture line proximal to the conidiophore apex, and (3) the unprecedented formation of digitate (finger-like) chlamydospores directly from primary conidia. To determine the taxonomic position and evolutionary relationships of this isolate, we conducted integrated morphological characterization and multilocus phylogenetic analyses utilizing 28S and ITS regions in combination with *RPB2* sequences. Our comprehensive molecular and morphological evidence supports the designation of this isolate as a novel genus phylogenetically sister to *Basidiobolus* and formally distinct within Basidiobolaceae.

## Materials and methods

### Isolation and morphological study

Decaying plant litter was collected from the Muriel B. Mundy Wildflower Garden (Cornell Plantations, Ithaca, NY, USA) in December 2005. To isolate these elusive ballistosporic fungi, we deployed a modified inverted plate technique: homogenized litter was suspended over semi-solidified corn meal agar (CMA), with an inverted potato dextrose agar (PDA) dish used to capture forcibly discharged propagules. Plates were incubated at 21 °C under ambient light. An extraordinary colony exhibiting Entomophthorales-like characteristics was subsequently isolated, subcultured, and cryopreserved (accession: ARSEF 7942). Morphological characterization rigorously followed King’s established techniques for *Conidiobolus* (King 1976a, 1976b), utilizing a Nikon Eclipse E600 microscope and a SPOT RT digital camera. Microstructures (mycelium, primary conidiophores, primary and secondary conidia, and resting spores) were examined on PDA colonies. Measurements of morphological structures (n ≥ 30 for each feature) were recorded, and observations were repeated using independent cultures.

### DNA extraction, PCR amplification and sequencing

Axenic mycelia were propagated on potato dextrose agar (PDA) overlaid with sterile cellophane and incubated at 25 °C for 7 d. The resulting vegetative biomass was harvested, flash-frozen in liquid nitrogen, and thoroughly pulverized utilizing a sterile micropestle. Genomic DNA was rigorously extracted following the benzyl chloride protocol (Zhu et al. 1994), resuspended in 50–100 μL of HPLC-grade water, and cryopreserved at –20 °C. Three key phylogenetic marker regions were amplified via PCR: the partial nuc 28S rDNA D1-D2 regions (28S) using the primer pair LR0R (5’-ACCCGCTGAACTTAAGC-3’) and LR5 (5’-TCCTGAGGGAAACTTCG-3’) (Vilgalys and Hester 1990); the RNA polymerase II second largest subunit (*RPB2*) utilizing novel primers gRPB2-7F (5’-ATGGGYAARCAGGCYATGGG-3’) and eRPB2-2500 (5’-AANGGWGTVGCRTCWCCTTC-3’) designed based on multiple sequence alignments of conserved regions across representative Basidiobolaceae and related zygomycete taxa for this study; biological replicates were performed for all PCR-based assays to ensure reproducibility, and the complete nuclear ITS1-5.8S-ITS2 region (ITS) employing primers ITS4 (5’-TCCTCCGCTTATTGATATGC-3’) and ITS5 (5’-GGAAGTAAAAGTCGTAACAAGG-3’) (White et al. 1990). Amplification of the LSU and ITS regions strictly adhered to established standard thermocycling protocols (White et al. 1990; Vilgalys and Hester 1990). For the *RPB2* locus, PCR was optimized in 50 μL reaction volumes containing 200 μM dNTPs, 1× Mg-free buffer, 2.5 mM MgCl_2_, 0.5 μM of each primer, 2% DMSO, 10–50 ng of template DNA, and 0.04 U/μL Taq polymerase (Promega). The specific thermocycling parameters for *RPB2* consisted of an initial denaturation at 95 °C for 5 min; 34 cycles of 94 °C for 1 min, 55 °C for 2 min, and 72 °C for 2 min; culminating in a final extension at 72 °C for 10 min. The resulting amplicons were meticulously purified using Qiagen QIAquick kits (Gel Extraction Kit for *RPB2*; PCR Purification Kit for 28S and ITS) and subsequently bidirectionally sequenced at the Cornell University Biotechnology Resource Center. All newly generated sequences essential to resolving this phylogenetic framework were deposited in GenBank (Table 1).

**Table 1:** Species used in the phylogenetic analyses.

**Species**	**Strain number**	**Host/Substrate**	**Country**	**GenBank accession numbers**		
				**ITS**	**28S**	***RPB2***
*Basidiobolus arizonensis*	CBS 149860	Dog	USA	OQ449342	OQ450351	N/A
*Basidiobolus haptosporus*	ARSEF 261	Human	Indonesia	EF392520	MH869969	EF392465
	ARSEF 6380	Soil and litter	UK	EF392528	EF392417	EF392472
	ARSEF 8307	N/A	USA	EF392529	EF392418	EF392473
	ARSEF 8309	N/A	USA	EF392531	EF392420	EF392475
*Basidiobolus heterosporus*	ARSEF 262	Oak leaf	USA	EF392521	EF392411	EF392466
	ATCC 16580 (T)	Plant debris	India	EF392536	EF392423	EF392478
*Basidiobolus magnus*	ATCC 15379 (T)	Plant debris	USA	EF392534	EF392425	EF392479
*Basidiobolus meristosporus*	CBS 931.73	Gecko dung	Ivory Coast	MT830914	MT831974	N/A
	CBS 930.73	Ant cemetery of Paltothyreus tarsatus	Ivory Coast	MT830915	MT831975	N/A
*Basidiobolus microsporus*	ARSEF 265	Lizard dung	USA	EF392523	DQ364202	DQ364212
	CBS 130.62 (T)	Lizard dung	USA	MH858122	MH869698	N/A
*Basidiobolus minor*	ATCC 16579 (T)	*Ficus* fruit	India	EF392535	EF392424	EF392480
*Basidiobolus omanensis*	CBS 146281 (T)	Human intravascular thrombus	Oman	MT830913	MT831973	N/A
	CBS 146282	Human urine	Oman	MT830912	MT831972	N/A
*Basidiobolus ranarum*	AFTOL-ID 301	N/A	N/A	AY997030	DQ273807	DQ302777
	CBS 538.63	Frog	Canada	MH858348	MH869969	N/A
*Batkoa apiculata*	ARSEF 3130	*Acyrthosiphon pisum*	USA	N/A	EF392404	EF392459
*Batkoa gigantea*	ARSEF 214	*Tipula paludosa*	Switzerland	N/A	JX242591	EF392433
*Batkoa major*	ARSEF 2936	*Empoasca fabae*	USA	N/A	EF392401	EF392457
*Conidiobolus brefeldianus*	ARSEF 452	Plant debris	USA	N/A	EF392382	N/A
*Conidiobolus coronatus*	AFTOL-ID 137	N/A	N/A	AY997041	AY546691	DQ302779
*Conidiobolus firmipilleus*	ARSEF 6384	Soil and litter	UK	N/A	JX242592	N/A
***Drechslerosporium cornellii***	**ARSEF 7942 (T)**	**Plant debris**	**USA**	**EF392537**	**EF392427**	**EF392482**
*Eryniopsis caroliniana*	ARSEF 640	*Tipula paludosa*	Switzerland	N/A	EF392387	EF392444
*Furia americana*	ARSEF 742	Diptera	Brazil	N/A	EF392389	EF392446
*Olpidium brassicae*	AFTOL-ID 633	N/A	N/A	AY997067	DQ273818	N/A
*Powellomyces* sp.	AFTOL-ID 32	N/A	N/A	AY997075	DQ273776	N/A
*Pandora kondoiensis*	CBS 642.92	N/A	N/A	N/A	JX242603	JX266788
*Schizangiella serpentis*	ARSEF 203	*Crotalus horridus*	USA	EF392538	EF392428	EF392481
	ARSEF 8000	*Elaphe obsoleta*	USA	EF392539	EF392426	EF392483
*Schizangiella* sp.	ARSEF 2237	*Elaphe* sp.	Germany	EF392540	EF392429	EF392484
*Zoophthora anglica*	ARSEF 396	*Agriotes sputator*	Switzerland	N/A	EF392379	EF392436

*AFTOL-ID, Assembling the Fungal Tree of Life, Department of Biology, Duke University (NC, USA); ARSEF, ARS Entomopathogenic Fungus Collection (Ithaca, U.S.A.). ATCC, American Type Culture Collection (Manassas, USA). CBS, Westerdijk Fungal Biodiversity Institute (Utrecht, The Netherlands). T = ex-type. The new species reported in this study is indicated in bold.

### Phylogenetic analyses

To establish a definitive evolutionary framework, we reconstructed a comprehensive phylogenetic tree incorporating molecular sequences from all sampled *Basidiobolus* species (Al-Hatmi et al. 2021). Reference nucleotide sequences spanning three critical loci (28S, ITS, and *RPB2*) were retrieved from the GenBank database (Table 1). Multiple sequence alignments were executed utilizing MAFFT v7 (Katoh et al. 2019). To ensure maximum phylogenetic robustness, ambiguously aligned regions were strictly eliminated employing the Gblocks online server using the default parameters (http://www.phylogeny.fr/one_task.cgi?task_type=gblocks).

For precise outgroup rooting, we selected *Olpidium brassicae* (AFTOL-ID 633) and *Powellomyces* sp. (AFTOL-ID 32). The highly curated alignments of the three loci were subsequently concatenated using SequenceMatrix v1.7.8 (Vaidya et al. 2011). This assembled multilocus dataset was deposited in TreeBase (https://treebase.org) under the submission ID S32144. Both maximum likelihood (ML) and bayesian inference (BI) algorithms were deployed to reconstruct the phylogeny. ML analyses were executed in IQ-TREE v2.3.6 (Nguyen et al. 2015), with the best-fitting nucleotide substitution model selected automatically by ModelFinder (Kalyaanamoorthy et al. 2017). BI analyses were conducted using MrBayes v3.2.7 (Ronquist et al. 2012), with the optimal nucleotide substitution models selected for each locus individually using Modeltest 3.7 (Posada and Crandall 1998). The Bayesian analyses utilized four simultaneous Markov Chain Monte Carlo (MCMC) chains across two independent runs, which were strictly terminated only once the average standard deviation of split frequencies conclusively dropped below the stringent 0.01 convergence threshold. The initial 25% of sampled trees from each run were discarded as burn-in, utilizing the remaining 75% to calculate robust posterior probabilities (PP). Finally, the resulting phylogenetic trees were visualized with FigTree v1.4.4 (Rambaut 2012) and aesthetically annotated for formal publication utilizing the iTOL web platform (www.itol.embl.de/).

## Results

### Phylogenetic analyses

**Figure 1.**
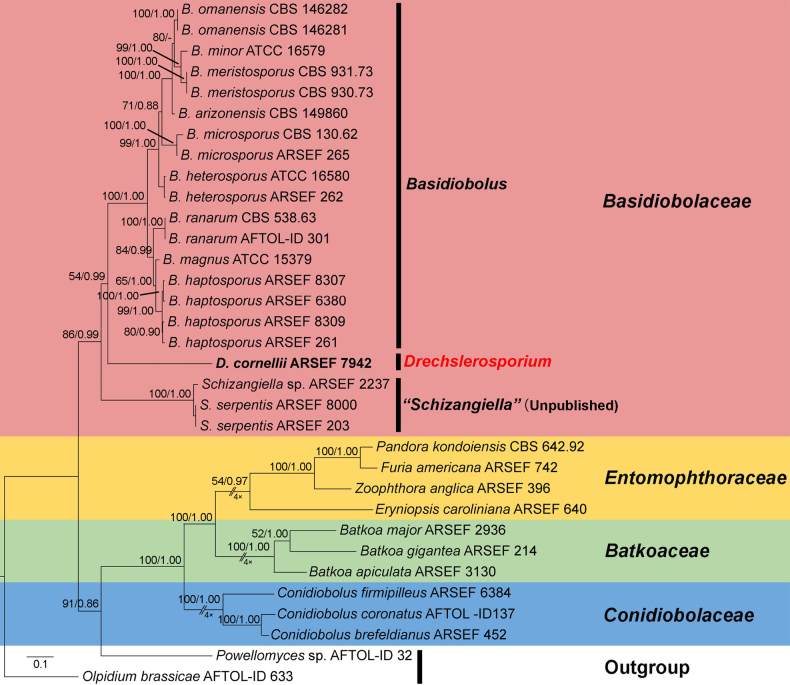
Phylogenetic tree of the family Basidiobolaceae based on 28S, ITS, and *RPB2* loci. *Olpidium brassicae* (AFTOL-ID 633) and *Powellomyces* sp. (AFTOL-ID 32) were used as outgroups. Maximum likelihood bootstrap values (≥ 50%) / Bayesian posterior probabilities (≥ 0.80) are indicated along each branch. The scale bar at the bottom left indicates the number of substitutions per site. New species is indicated in bold and new genus is highlighted in red.

The concatenated dataset (28S, ITS, and *RPB2*) comprised 2,136 nucleotide positions (894, 519, and 723 sites, respectively). For ML analyses, the best-fitting substitution models were GTR+F+R4 for the 28S and *RPB2* loci, and TPM3u+F+R3 for the ITS locus. For BI analyses, the optimal models were TrNef+G for 28S and ITS, and TIMef for *RPB2*. Bayesian analyses (two million generations, sampled every 100) achieved robust convergence with a final average standard deviation of split frequencies of 0.001256. Topologies generated by maximum likelihood (ML) and bayesian inference (BI) were highly congruent; thus, the ML phylogram is presented (Fig. 1).

Phylogenetic reconstruction unequivocally resolved all recognized *Basidiobolus* species into a strictly monophyletic clade (support: 100/1.0). Crucially, our focal isolate ARSEF 7942 segregated entirely from the *Basidiobolus* complex, forming a distinct evolutionary lineage (support: 54/0.99). Furthermore, the unpublished genus “*Schizangiella*” (Humber 2012; Gryganskyi et al. 2012; Nie et al. 2025) formed a sister lineage to ARSEF 7942 (support: 86/0.99). Although the bootstrap value for the ARSEF 7942 lineage falls below the conventional 70% threshold, its monophyly is corroborated by congruent topologies under alternative inference methods (ML and BI), combined morphological distinctiveness, and absence of shared synapomorphies with *Basidiobolus* species. Collectively, the above evidence supports recognizing ARSEF 7942 as a novel genus in Basidiobolaceae.

### Taxonomy

#### Basidiobolaceae

Taxon classificationFungiBasidiobolalesBasidiobolaceae

Claussen, Syllab. Pflanzenfam., Edn 9 & 10: 45 (1924) emend.

EF69996E-9E5F-5555-BCD3-847817D31AB6

80514

##### Type genus.

*Basidiobolus* Eidam, Beitr. Biol. Pflanz. 4: 194. 1886. *Drechslerosporium* B. Huang & Y. Nie, gen. nov., this article.

##### Description.

Vegetative cells uninucleate, septate mycelium or yeast-like. Mitosis with nuclear membrane fragmentation, tiny, condensed chromosomes aligned on a central metaphase plate (often within nucleolus), barrel-shaped spindle. Conidiophore simple with bulbous apical swelling. Conidia uninucleate, globose, with conical basal papilla, discharged forcibly by circumscissile rupture of subconidial swelling, its upper part carried with conidium. Discharged conidia retain prominent conidiophore appendages after circumscissile rupture of the apical swelling. Secondary conidia arising from primary conidia, similar to the primary ones. Capilliconidia elongate, often curved, on capillary conidiophore, passively dispersed. Resting spores usually zygospores, homothallic, thick bilayered wall; germinate by direct formation of a germ conidium (elongate, passively dispersed). Resting spores of digitate chlamydospores develop directly from primary conidia.

##### Note.

In this article, we revised the family of Basidiobolaceae to include *Basidiobolus* and the newly established genus *Drechslerosporium*. Meanwhile, the diagnosis of Basidiobolaceae has been updated to include two defining morphological features of the new genus: (1) discharged conidia retaining prominent conidiophore appendages after circumscissile rupture of the apical swelling, and (2) digitate chlamydospores developing directly from primary conidia. Particularly, although “*Schizangiella*” is recovered within Basidiobolaceae in phylogenetic analyses, it lacks formal publication. Consequently, it is excluded from the present taxonomic revision of the family.

#### Drechslerosporium

Taxon classificationFungiBasidiobolalesBasidiobolaceae

B. Huang & Y. Nie gen. nov.

4CA93CA2-0F04-58FA-AA9B-5A707F4294C9

862732

##### Etymology.

In honor of mycologist Charles Drechsler who described many new American species of *Conidiobolus* and *Basidiobolus*.

##### Type species.

*Drechslerosporium cornellii* B. Huang & Y. Nie.

##### Description.

Vegetative growth as septate mycelium with uninucleate cells. Nuclei large, with a prominent central nucleolus, not staining with aceto-orcein. Conidiophore simple with a swollen apex subtending the solitary conidium. Primary conidia unitunicate, uninucleate, bearing the empty remains of distal parts of the propulsive conidiophore, which are observed remaining attached to the conidium. Forcible discharge by backward ejection of conidiophore contents upon circumscissile rupture of the apical distention of the conidiophore. Secondary conidia produced from the discharged primary conidia, and of two types: 1) of reduced size but similar to primary conidia in shape and forcibly discharged in a similar manner from a short conidiophore; 2) elongated capilliconidia with an apical beak covered distally with adhesive material. Primary conidia occasionally give rise to a single conidiophore, bearing a digitate chlamydospore terminally. Zygospores unknown.

#### Drechslerosporium cornellii

Taxon classificationFungiBasidiobolalesBasidiobolaceae

B. Huang & Y. Nie sp. nov.

BD1F4560-8716-5E7C-8329-2666807B02BC

862733

Fig. 2

##### Etymology.

Named in honor of Ezra Cornell (1807–1874), founder of Cornell University.

**Figure 2.**
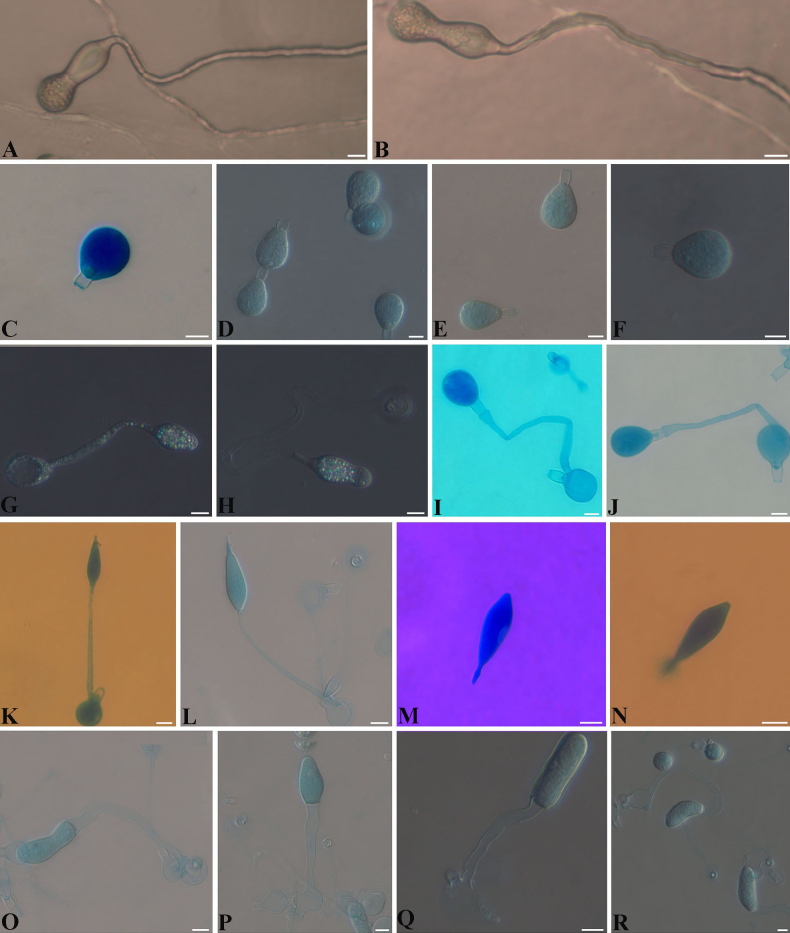
*Drechslerosporium cornellii* ARSEF 7942. **A, B**. Mycelia and primary conidiophore with a propulsive distention at the tip; **C, D, E, F**. Conidia with an apical protrusion at the base; **G, H, I, J**. Secondary conidiophores bearing a single secondary conidium; **K, L**. Secondary conidiophores bearing a single capilliconidium; **M, N**. Elongated capilliconidia; **O–R**. A single conidiophore bearing a digitate terminal chlamydospore. Scale bars: 10 μm (**A–R**).

##### Typification.

USA. New York: Ithaca, Cornell University campus, Muriel B. Mundy Wildflower Garden of the Cornell Plantations, 42°26'59.9"N, 76°28'9.1"W, 15 Nov 2005, isolated from plant detritus by *B. Huang* (holotype ARSEF 7942, deposited at the USDA-ARS Collections of Entomopathogenic Fungal Cultures, Fig. 2). GenBank: EF392537 (ITS); EF392427 (28S); EF392482 (*RPB2*).

##### Description.

Colonies grow slowly at 25 °C in 3 d. At first pale brown, becoming fuzzy and white in the central region after production of primary conidia. Reverse usually pale brown, changing with age to dark. Odor absent. Mycelium colorless, usually with little or no aerial growth, at the margin of an expanding mycelium often terminating in a cell 95.0–344.0 × 6.7–8.2 μm. forming many protuberances (immaturity branches) in a hyphal segment at first; and continue swelling to forming cells with various shape. Primary conidiophores arising from a hyphal segment, colourless, unbranched, mostly 5.4–9.1 μm wide, extending often 91–237 μm into the air, terminally inflated into a propulsive distention that often is elongated ellipsoidal, 27.1–38.3 μm long, 14.6–23.5 μm wide, and bears one conidium, and forcibly shooting it off which breaks in the fore part of a propulsive distention; Conidia colorless, subspherical to obovoid, with an apical protrusion at the base, 22.2–36.3 × 17.3–29.2 μm, appended with the empty membranous remains of distal parts of propulsive distention (never separated from conidium), mostly cylindrical, 4.9–9.9 × 4.9–7.4 μm. Secondary conidia two types: 1) similar to primary conidia in shape, violent manner of discharge and appending with empty membranous remains of distal parts of propulsive distention (never separated from conidium), 20–25.6 × 15.6–20 μm; 2) elongated capilliconidia, mostly prolate ellipsoidal, straight, 11.1–17.0 μm in greatest width, 43.2–56.8 μm in total length inclusive of an apical beak that usually is 9.1–12.4 μm long and is covered distally with adhesive material. Occasionally, primary conidia give rise to a single conidiophore under suboptimal environmental conditions within 3–5 d, bearing a digitate chlamydospore terminally; 38–53 × 12.2–19 μm. Zygospores not observed.

## Discussion

Morphologically, *Drechslerosporium* shares foundational synapomorphies with *Basidiobolus*, notably the production of uninucleate primary conidia, congruent conidial wall ultrastructure, fundamentally homologous primary conidiophore architecture, and the capacity to generate capilliconidia. However, it displays notable evolutionary divergences in its conidial discharge mechanism — an important morphological trait that has historically been widely recognized as a key diagnostic criterion at the generic level among allied fungi. Macroscopically, the darkly pigmented colonies of *Drechslerosporium* on PDA contrast markedly with the typically hyaline mycelia of all known *Basidiobolus* species. Furthermore, the ontogeny of digitate chlamydospores arising from conidia represents an autapomorphy unrecorded in any allied taxa.

**Figure 3.**
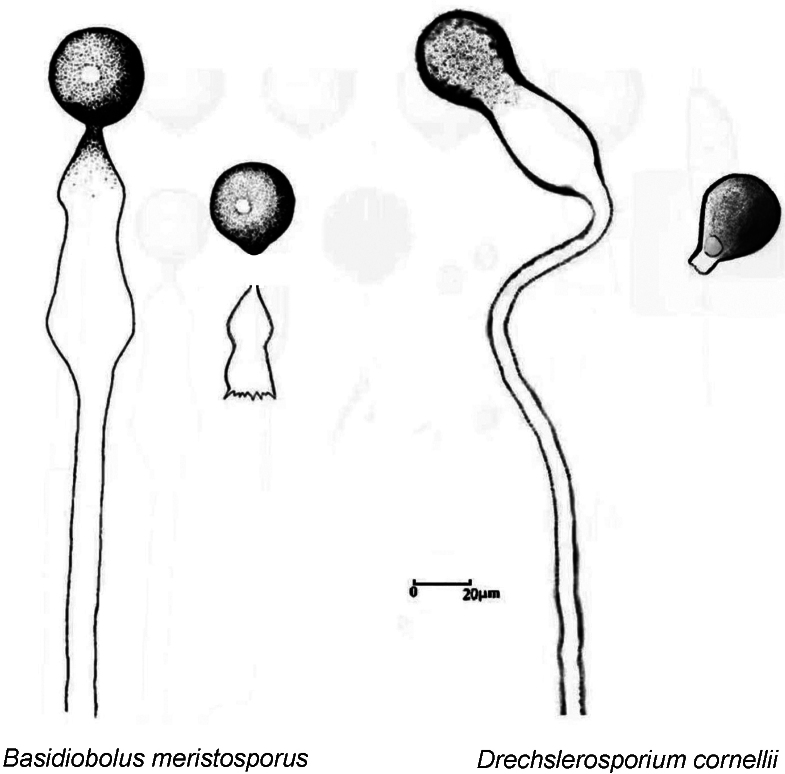
The developmental origin of primary conidiophores and the pattern of septation during primary conida formation between *Basidiobolus meristosporus* and *Drechslerosporium cornellii*.

The biomechanics of conidial discharge in this novel genus represent a distinctive functional adaptation. In typical *Basidiobolus* species, the conidiophore dehisces cleanly at a circumscissile zone strictly at the base of the subtending apical vesicle, unleashing a pressurized blast of cytoplasmic contents that violently expels the conidium. During flight, these conidia overwhelmingly separate from the propulsive remnants of the conidiophore apex via the eversion of a basal papilla. This classical explosive mechanism is widely defined as the “sporophore rocket”. In dramatic contrast, the circumscissile rupture in *Drechslerosporium* occurs anomalously higher, closer to the top of the apical vesicle, forcing the discharged conidium to permanently retain conspicuous, membranous remnants of the conidiophore apex (Fig. 3). Consequently, we designate this unprecedented evolutionary innovation as a “modified sporophore rocket,” distinctly lacking the afterburner effect of papillar eversion during its trajectory. Anatomically, the conidium of *Drechslerosporium* conspicuously lacks a true columella; instead, it is delimited solely by a basal septum, irrevocably tethering it to the propulsive remnants post-discharge.

Functionally, the highly specialized discharge mechanism of *Drechslerosporium* convergently resembles that of *Ballocephala*, where discharged propagules similarly carry the ruptured apex of the conidiogenous cell. However, this superficial mechanical similarity belies profound structural disparities: the conidiophore of *Drechslerosporium* is rigorously simple and unbranched, whereas *Ballocephala* relies on a highly complex conidiophore bearing numerous short lateral branches, each terminating in a solitary conidium. Notably, *Ballocephala* has been excluded from the Entomophthorales and transferred to the Kickxellomycotina (Hibbett et al. 2007).

Phylogenetically, our comprehensive multilocus framework unequivocally positions *Drechslerosporium* as a sister lineage to *Basidiobolus*, establishing a deeply divergent and profoundly distinct evolutionary clade strictly within the boundaries of the Basidiobolaceae. This topological discovery absolutely necessitates a radical redefinition of the entire family to accommodate both genera, brilliantly and definitively resolving the historical taxonomic inconsistencies that have long plagued this formerly monogeneric classification.

While this study formally establishes the taxonomic status of *Drechslerosporium*, it highlights clear avenues for future research within this enigmatic group: (1) evaluating the taxonomic weight of digitate chlamydospores as a genus-level diagnostic criterion within the Basidiobolaceae, and (2) comprehensively mapping the global ecology, geographic distribution, and untapped species-level diversity within this genus. Additionally, our multilocus phylogeny isolates the undescribed taxon “*Schizangiella*” as a distinct, deeply divergent lineage within the Basidiobolaceae, resolved close to *Drechslerosporium*. Given its phylogenetic placement and genetic distance, “*Schizangiella*” warrants formal description as an independent genus. Consequently, future phylogenomic investigations incorporating these underrepresented lineages will be essential to resolve the macroevolutionary patterns and diversification history of this redefined family.

## Supplementary Material

XML Treatment for Basidiobolaceae

XML Treatment for Drechslerosporium

XML Treatment for Drechslerosporium cornellii

## Additional information

### Artificial Intelligence (AI) use

The authors accept full responsibility for the content of the manuscript, including the disclosure of any use of AI.

No AI tools were used in the preparation of this manuscript.

### Author ORCIDs

Y. Nie https://orcid.org/0000-0001-8964-1661

B. Huang https://orcid.org/0000-0001-6032-7396

### Data availability

Sequences (ITS, 28S, and *RPB2*) of the new species are available in GenBank under the accession numbers listed in Table 1. The alignment used for analyses is deposited in TreeBase (https://treebase.org) under the submission ID S32144, and it is also available as a supplementary file.

## Supplementary materials

Supplementary material 1 Alignments for phylogen Data type rar https://binary.pensoft.net/file/1709666This dataset is made available under the Open Database License (http://opendatacommons.org/licenses/odbl/1.0/). The Open Database License (ODbL) is a license agreement intended to allow users to freely share, modify, and use this Dataset while maintaining this same freedom for others, provided that the original source and author(s) are credited. Yong Nie, Bo Huang

Supplementary material 2 BI tree Data type rar https://binary.pensoft.net/file/1709667This dataset is made available under the Open Database License (http://opendatacommons.org/licenses/odbl/1.0/). The Open Database License (ODbL) is a license agreement intended to allow users to freely share, modify, and use this Dataset while maintaining this same freedom for others, provided that the original source and author(s) are credited. Yong Nie, Bo Huang
